# Photocatalytic and Photothermal Antimicrobial Mussel-Inspired Nanocomposites for Biomedical Applications

**DOI:** 10.3390/ijms241713272

**Published:** 2023-08-26

**Authors:** Luis F. Soto-Garcia, Ingrid D. Guerrero-Rodriguez, Luu Hoang, Samantha Lauren Laboy-Segarra, Ngan T. K. Phan, Enrique Villafuerte, Juhyun Lee, Kytai T. Nguyen

**Affiliations:** Department of Bioengineering, The University of Texas at Arlington, Arlington, TX 76010, USA

**Keywords:** antimicrobial, photocatalytic, photothermal, nanocomposites, polydopamine, titanium oxide

## Abstract

Bacterial infection has traditionally been treated with antibiotics, but their overuse is leading to the development of antibiotic resistance. This may be mitigated by alternative approaches to prevent or treat bacterial infections without utilization of antibiotics. Among the alternatives is the use of photo-responsive antimicrobial nanoparticles and/or nanocomposites, which present unique properties activated by light. In this study, we explored the combined use of titanium oxide and polydopamine to create nanoparticles with photocatalytic and photothermal antibacterial properties triggered by visible or near-infrared light. Furthermore, as a proof-of-concept, these photo-responsive nanoparticles were combined with mussel-inspired catechol-modified hyaluronic acid hydrogels to form novel light-driven antibacterial nanocomposites. The materials were challenged with models of Gram-negative and Gram-positive bacteria. For visible light, the average percentage killed (PK) was 94.6 for *E. coli* and 92.3 for *S. aureus*. For near-infrared light, PK for *E. coli* reported 52.8 and 99.2 for *S. aureus*. These results confirm the exciting potential of these nanocomposites to prevent the development of antibiotic resistance and also to open the door for further studies to optimize their composition in order to increase their bactericidal efficacy for biomedical applications.

## 1. Introduction

Pathogenic bacteria have long posed a serious threat to human health, causing potentially deadly infectious diseases in addition to hindering clinical treatments and healthcare [1,2,3,4]. Historically, antibiotics have been used to treat bacterial infections, with the discovery of penicillin, in particular, helping to fight many diseases [2,5]. However, even aside from the undesirable side effects that antibiotics can cause [1,2,6], including neurotoxicity [7], their overuse is reportedly one of the main causes of the development of bacterial antibiotic resistance (AR) [7,8,9], such as methicillin-resistant *S. aureus* [10,11,12]. In 2019, there was a worldwide estimate of 1.27 million deaths attributable to major AR syndromes, including skin and subcutaneous infections [13]. Hence, impeding the evolution of AR species via the use of alternative strategies to kill and/or arrest bacterial growth is a challenge that is being tackled with different approaches.

One way to undertake infection without the use of antibiotics is the inhibition of quorum sensing, which involves blocking the production of bacterial signal molecules to prevent biofilm formation [14,15,16,17]. Potential inhibitors include antibodies against bacterial peptides like AP4, which successfully blocked *S. aureus*-induced dermal injuries in mice [18,19]. However, its narrow spectrum of activity, possible unwanted effects against beneficial bacteria in the body, and inability to actually kill pathogens [18] may present drawbacks to its application [14]. Another potential alternative is phage therapy, in which viral bacteriophages are targeted to infect and kill specific bacteria by lysis without harming native cells [14,15,16]. As early as 1921, bacteriophages were described as effective against staphylococcal skin furuncles [20], and in 2016, a five-phage cocktail against *A. baumannii* was successfully used to heal severely infected wounds in mice [21]. However, there remain safety concerns over post-treatment phage clearance and the purity of prepared phages [14], as well as issues with how phage-resistant bacterial strains can quickly develop [14,16,17].

Another promising approach is the development of antimicrobial biomaterials, such as hydrogels, nanoparticles, or nanocomposites [22]. For instance, cationic hydrogels with demonstrated capabilities against *S. aureus* and *E. coli* have been recently developed for wound healing therapy, with promissory results in a diabetic rat model [23]. In another case, polydopamine-coated silver nanoparticles were synthesized and found to possess an antimicrobial effect at 12.5 µg/mL [24]. Besides these positive results, the combination of antibacterial hydrogels and nanoparticles with unique properties and mechanisms may create synergistic effects to enhance the performance of the resulting compounds, also known as nanocomposites. Among those special properties is photoresponsiveness, the characteristic of a material to present a physical response to light irradiation. This phenomenon occurs when the electromagnetic waves of light interact with the electronic matrix of the material, forming electron-hole pairs which stimulate a variety of effects such as electron transfer to generate electrical current, release of superficial molecules, heat generation, or induction of chemical reactions [25]. Among them, the generation of thermal energy and the ability to produce or catalyze chemical reactions are particularly important when creating antimicrobial materials.

One such material, which has been explored due to its light-induced heat generation capabilities, is polydopamine (PDA) [26,27,28], a biomimetic polymer derived from the oxidation of dopamine (DA) [1,2,29]. On its own, PDA exhibits advantages such as elevated biocompatibility [30,31] and high availability of functional groups, such as catechols, amines, imines, and carboxyl groups, which allow for the conjugation of a variety of bio molecules containing amino, aldehyde, or sulfhydryl groups, including the formation of pH-sensitive reversible Schiff base bonds [27,32,33,34]. As a result, PDA can be tightly attached to the surface of nanomaterials and also work as a bridge or coating to facilitate posterior modifications. Hence, in addition to their photoresponsive characteristics, polydopamine has the potential to be used as a platform to carry different chemicals or biomolecules for an efficient stimulus-responsive drug delivery system. Moreover, PDA also presents a remarkable photo-responsive property called photothermal effect, which is the generation of thermal energy by the relaxation of excited electrons [25]. This transducer effect increases the temperature at the vicinity of the PDA surface, inducing the degradation of the bacterial membrane to create an antimicrobial effect [35,36,37]. For example, PDA has been incorporated into a silver-sodium lignin sulfonate nanoparticle-containing hydrogel to exert an effective in vitro photothermal effect against both *E. coli* and *S. aureus* under NIR light irradiation at 808 nm [38]. Under similar conditions, PDA has also been used as a coating for ferrous sulfide nanoparticles [39] and gold-hydroxyapatite nanoparticles for the photothermal treatment of infected skin defects [40]. Moreover, PDA reportedly exerts additional activity against the bacterial wall via electrostatic interactions, chelation of proteins, catechol-mediated chelation of bacterial metals, and ROS production [1,2].

Furthermore, even though its carbon-based nature does not allow PDA to show photocatalytic activity, it can still present synergistic interactions with photocatalytic metal oxides (PMO) by expanding the amount of available π–π* electron transfer channels, which results in enhanced generation of electron-pair holes under light irradiation [27]. For instance, PDA has been compounded with carbon nitride, silver nanoparticles, and chitosan to create a photocatalytic film with antimicrobial and wound healing properties [41]. Besides silver, another interesting metallic compound is titanium dioxide (TiO_2_) [42,43], which by itself exhibits extraordinary photocatalytic properties. When exposed to ultraviolet (UV) light [44], TiO_2_ nanoparticles can produce electron-hole pairs, which react chemically with oxygen and environmental moisture to form reactive oxygen species (ROS) [45,46] able to kill bacteria [47,48,49] or to degrade harmful components in wastewater [50]. However, for human health applications, this same fact is the main drawback of TiO_2_ nanoparticles because of the inherent risk of DNA damage under UV light exposure. To overcome the limited range of spectral response and quantum efficiency [51] of TiO_2_, a surface-coating strategy based on oxidative polymerization of PDA has proven its efficacy [45,51,52] to extend the photocatalytic effect of TiO_2_ into the visible light range. This permits the PDA-TiO_2_ compound to exert a photocatalytic, ROS-driven antimicrobial effect under riskless illumination conditions. Hence, the potential of nanotechnology to create safer photoresponsive biocidal or bacteriostatic materials to prevent AR development is remarkable [53].

In this research work, we explored the synergy between PDA and TiO_2_ to create photoresponsive antibacterial nanoparticles/nanocomposites activated by visible and NIR light. We used an in situ oxidation polymerization method [52,54] to synthesize PDA-coated TiO_2_ nanoparticles (PDA PMO) and evaluated their photocatalytic and photothermal capacities to reduce or eliminate inoculums of bacterial models *E. coli* (Gram-negative) or *S. aureus* (Gram-positive). In addition, we selected the mussel inspired hydrogel (MIH) hyaluronic acid-dopamine (HADA) as the vehicle. MIHs are biomaterials that mimic the naturally occurring wet adhesion of mollusks, such as blue mussels (Mytilus edulis) [33,55], and their main mechanism of action is based on the molecular interactions of catechols like dopamine. Furthermore, the polymeric backbone of HADA is hyaluronic acid (HA), a highly binding, bioactive and mucoadhesive polymer [56,57] present in all vertebrates [58]. HA is widely used in a variety of biomedical applications including drug delivery [59] and wound healing [60] due to its influence in the dermal metabolism [61]. Moreover, HADA and other catechol-modified HA biomaterials have found an important number of applications in wound healing [62,63], tissue engineering [64], and drug delivery [65]. We previously reported the synthesis of HADA and its incorporation with PDA nanoparticles to form a nanocomposite (HADA PDA) with potential use as a biodegradable bio-adhesive material [66]. Hence, as proof of concept, we used a mix of HADA and PDA PMO nanoparticles to form the photoresponsive antimicrobial nanocomposite HADA PDA PMO. In this article, we report our preliminary work on the evaluation of HADA PDA PMOs ability to exert antimicrobial activity in vitro under visible or NIR light irradiation.

## 2. Results

### 2.1. Characterization of PDA PMO

**Figure 1:**
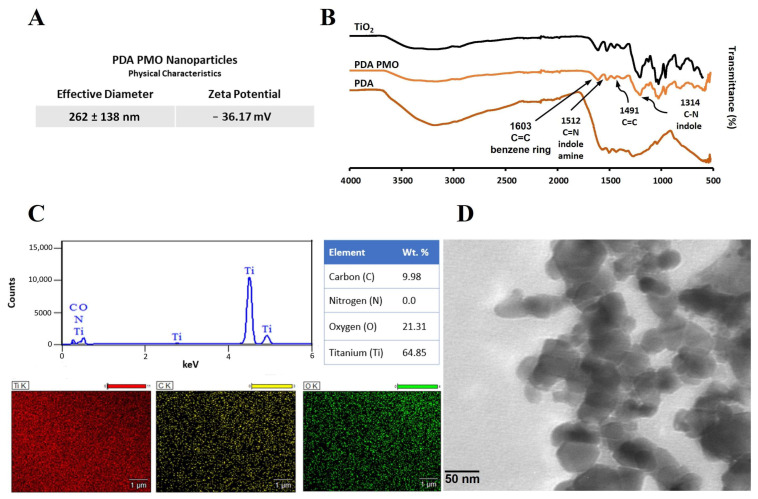
Characterization of PDA PMO nanoparticles. (**A**) Hydrodynamic size and zeta potential. (**B**) FTIR of nanoparticles and their components. (**C**) EDS spectra along with composition and SEM images. (**D**) TEM image.

Photocatalytic PDA PMO nanoparticles were synthesized and characterized as described in Section 4.2 and Section 4.3. Dynamic light scattering (DLS) analysis revealed that PDA PMO nanoparticles have an average effective diameter of 262 ± 138 nm and a PDI (polydispersity index) of 0.196. Additionally, the phase analysis light scattering (PALS) study resulted in a zeta potential of −36.17 mV (Figure 1A).

**Figure 2:**
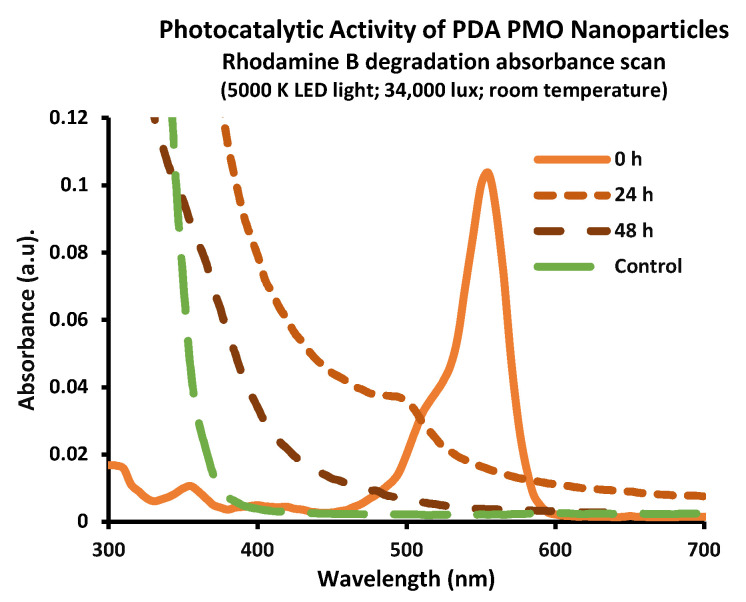
Photocatalytic activity of PDA PMO nanoparticles. The absorption scan confirmed the generation of reactive oxygen species resulting in 96% degradation of RhB (1 ppm, 48 h) The presence of a hypsochromic shift in the absorbance peak has been reported as evidence of advanced oxidative processes.

The Fourier transform infrared spectroscopy (FTIR) analysis confirmed the coating of PDA on TiO_2_. The spectrum exhibited an ample peak around 3400 cm^−1^, which relates to the O−H in the catechol group. Similarly, the peak at 1491 cm^−1^ is attributed to the C=C group characteristic of PDA, the peak at 1314 cm^−1^ is assigned to the C−N stretching of indole ring, the one at 1512 cm^−1^ to C=N of indole amine, and the one at 1603 cm^−1^ to carbon double bonds of benzene [51,67,68] (Figure 1B). Moreover, the energy-dispersive X-ray spectroscopy (EDS) spectrum confirmed the presence of titanium (64.8%), oxygen (21.3%), carbon (9.9%), and traces of nitrogen—all the components of PDA PMO (Figure 1C). Furthermore, high resolution transmission electron microscopy (HR-TEM) images show PDA PMO nanoparticles presenting an oblong morphology (Figure 1D). The photocatalytic capacity of PDA PMO nanoparticles irradiated with visible light was assessed as a function of the oxidative degradation of rhodamine B (RhB), as described in Section 4.3. Initial results from the absorbance scan revealed a peak in absorbance at 555 nm (Figure 2), in agreement with the published literature [69,70]. The absorbance values at this wavelength were selected to calculate the total percentage of RhB degradation which resulted in a 96% reduction after 48 h. Moreover, as reported in the literature [54,70,71], an hypsochromic shift from 555 to 498 nm (Figure 2) was observed in the 24 h scan, thus confirming the capability of PDA PMO to create electron-hole pairs for subsequent generation of ROS.

### 2.2. Characterization of HADA Hydrogel and HADA PDA PMO Nanocomposites

**Figure 3:**
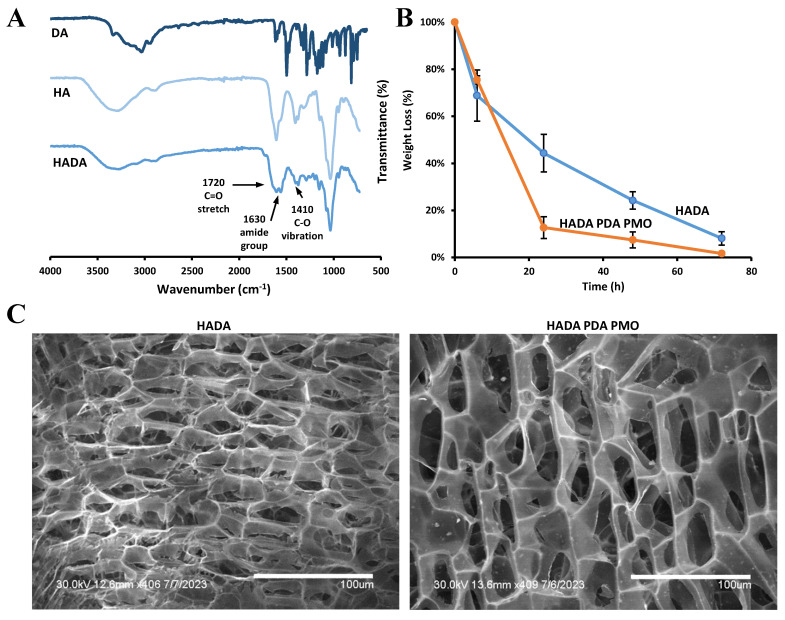
Characterization of HADA hydrogel and HADA PDA PMO nanocomposites. (**A**) FTIR of HADA and its components. (**B**) Degradation profiles of HADA and HADA PDA PMO. (**C**) SEM images of HADA hydrogel and nanocomposites.

HADA hydrogel was prepared and characterized as described in Section 4.4. The ultraviolet-visible spectroscopy (UV-Vis) study determined the amount of dopamine conjugated onto the backbone of hyaluronic acid as 13.3 ± 1.4%. The FTIR spectra presented peaks at 1720 cm^−1^ (C=O stretching), 1630 cm^−1^ (amide group), and 1410 cm^−1^ (C−O vibration) [72], thus confirming the grafting of DA to form HADA (Figure 3A). The biodegradation profiles of the HADA hydrogel and HADA PDA PMO nanocomposite in the presence of hyaluronidase both demonstrated about 90% weight loss in a period of 72 h (Figure 3B). The morphology of the hydrogel and the nanocomposite was observed using scanning electron microscopy (SEM) and revealed a similar structure in both cases. The average pore sizes were 22.8 ± 10.7 microns for HADA and 20.3 ± 4.7 microns for HADA PDA, both measured horizontally. Calculations of pore size were carried out using Image J 1.53 and scaled, respectively, to each image. (Figure 3C).

### 2.3. Cytocompatibility of Nanoparticles, Hydrogels, and Nanocomposites

**Figure 4:**
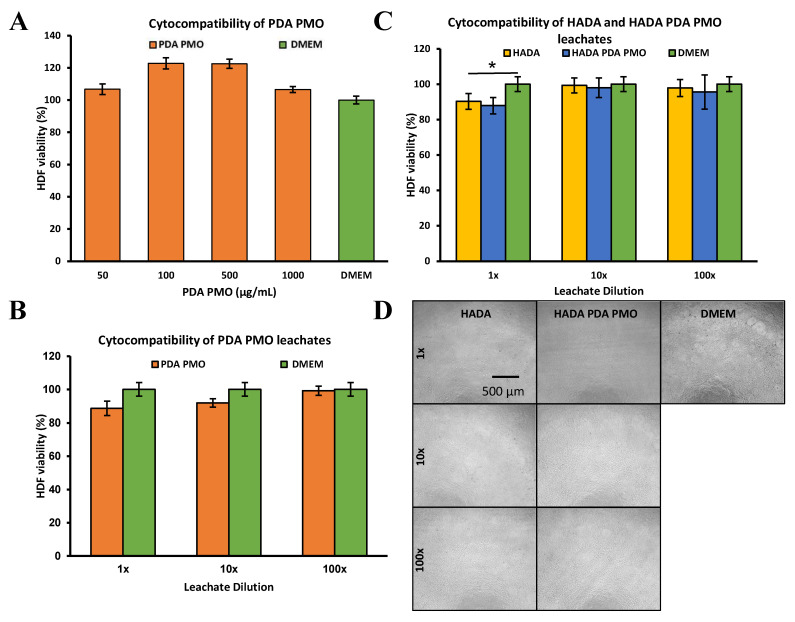
In vitro 72 h cytocompatibility studies on adult human dermal fibroblast HDFa. (**A**) HDFa treated with different concentrations of nanoparticle suspensions. (**B**) HDFa exposed to dilutions of leachates obtained from PDA PMO nanoparticles. (**C**) Hydrogel and nanocomposite leachates at various dilutions used to treat HDFa for 72 h. The asterisk (*) denotes a significant difference (*p* < 0.05). (**D**) Images of HDFa after 72 h exposure to nanocomposite and hydrogel leachates. Cells exposed to complete DMEM are the positive control.

The cell compatibility of PDA PMO nanoparticles, PDA PMO HADA hydrogel, and HADA PDA PMO nanocomposite was assessed using adult human dermal fibroblast (HDFa) cells, as described in Section 4.6. The compatibility study of PDA PMO suspensions at a range from 50 to 1000 µg/mL in complete media showed no adverse effect on cells after 72 h of exposure (Figure 4A).

Moreover, leachates from PDA PMO diluted in a range from 1 to 100× in complete media presented no toxicity issues with HDFa cells after 72 h of incubation (Figure 4B). Furthermore, the HADA hydrogel and HADA PDA PMO nanocomposite leachates diluted in the range of 1 to 100× with complete media exhibited values above 80% for all dilutions (Figure 4C), thus demonstrating adequate cytocompatibility in accordance with the International Standard ISO 10993-5:2009 Biological evaluation of medical devices—Part 5: Tests for in vitro cytotoxicity. Images of HDFa exposed to HADA and HADA PDA PMO leachates confirmed the last result (Figure 4D). In all of these experiments, complete media served as the positive control. Titanium dioxide was not included in cytocompatibility studies since its detrimental effects to human cells in vitro are well documented. For instance, researchers found that HDFa, cultured under a relatively low concentration of TiO_2_ nanoparticles (100 µg/mL), presented arrested collagen contractibility and reduced mobility, proliferation, and cellular area [73,74]. Another study using a ten-fold lower level of TiO_2_ nanoparticles on human epithelial cells also demonstrated cytotoxic effects, such as altered morphology and damaged DNA [75]. Moreover, previous in vivo studies in our group observed evidence of TiO_2_ nanoparticle toxicity to lung tissues, including cell infiltration, septal thickening, and collapsed airways [76]. Hence, considering these issues and its reduced antimicrobial performance under visible light, no experiments with TiO_2_ were deemed necessary.

### 2.4. Antimicrobial Studies

**Figure 5:**
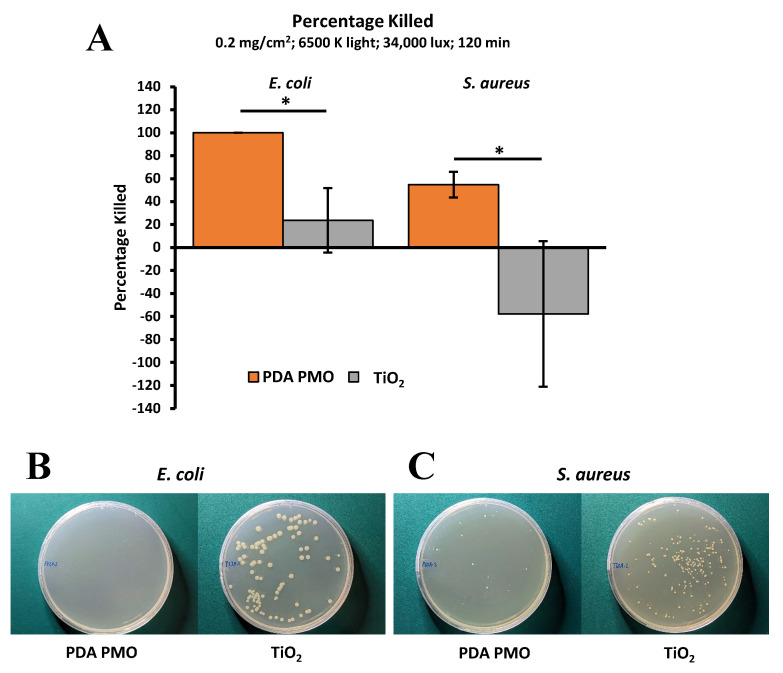
Comparison of antimicrobial performance of PDA PMO and TiO_2_ nanoparticles under visible light irradiation at room temperature. (**A**) PDA PMO kills 100% of *E. coli* and 54.6 ± 11.2% of *S. aureus* in 120 min, compared to 23.6 ± 28.0 and −57.8 ± 63.2%, respectively, for TiO_2_. The asterisk (*) denotes a significant difference (*p* < 0.05). (**B**). Images of *E. coli* after treatment with PDA PMO or TiO_2_. (**C**). Similar images for *S. aureus*.

The antimicrobial efficacy of TiO_2_ nanoparticles was compared against that of PDA PMO nanoparticles following the procedures described in Section 4.7. Results demonstrated that PDA PMO attained a higher percentage killed (PK) against *S. aureus* or *E. coli* as compared to TiO_2_ (Figure 5A,B), whose performance versus the latter was nominal. Moreover, in the case of *S. aureus*, TiO_2_ actually promoted bacterial growth, as shown by the negative PK value (Figure 5A,C). Hence, considering its lackluster performance, TiO_2_ was not considered for further experimentation.

**Figure 6:**
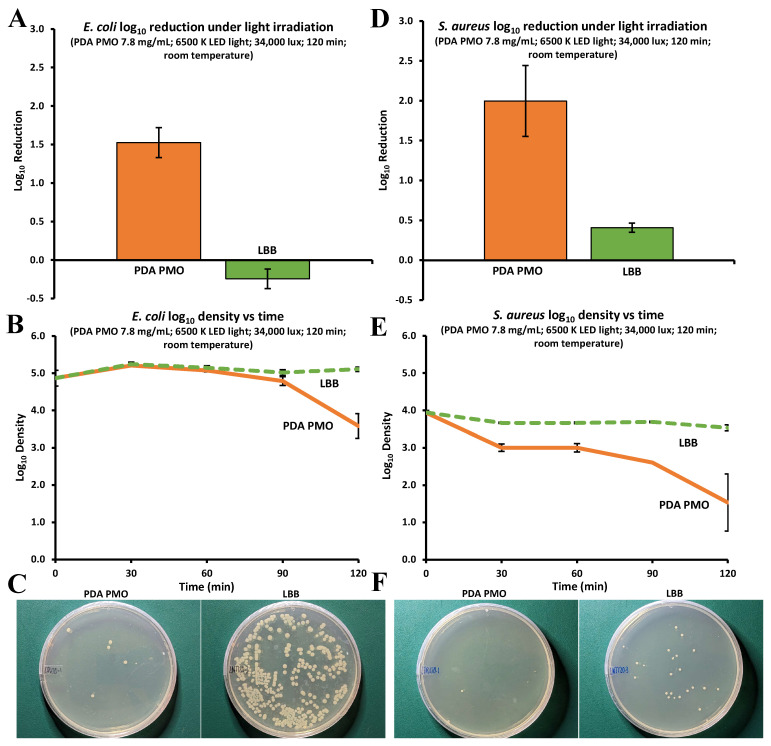
Antimicrobial activity of PDA PMO nanoparticles against *E. coli* and *S. aureus*. (**A**) *E. coli* log_10_ reduction after 120 min of light exposure compared to LB broth (negative control). (**B**) *E. coli* growth kinetics as log_10_ of bacterial density over a 120 min period. (**C**) Images of *E. coli* agar plates after treatment. (**D**–**F**) are similar graphs/images for *S. aureus*.

The antibacterial characteristics of PDA PMO nanoparticles, HADA hydrogels, HADA PDA PMO nanocomposites, and their components HA and DA were determined following the procedures described in Section 4.7. The photocatalytic antimicrobial activity of PDA PMO was determined under visible light irradiation produced by a commercial LED lamp. Results for *E. coli* show 1.53 ± 0.34 log_10_ reduction in bacterial population after 120 min (Figure 6A) with 94.3 ± 3.1% killed (PK). Moreover, the kinetic profile of the bacterial population presented an initial log_10_ value of 4.87 ± 0.37, which increased slightly during the first 30 min, followed by a decline to finally reach 3.58 ± 0.57 logs at 120 min (Figure 6B). Images of plated samples show the final result (Figure 6C). Similarly, data for *S. aureus* show a 2.00 ± 0.77 log_10_ reduction (Figure 6D). In contrast, the kinetic profile of the log_10_ bacterial population presents a decline from the initial value of 3.94 ± 0.10 until minute 30, when it remains stable until minute 60. Then, the population continues declining until it reaches a final value of 1.53 ± 1.33 logs at 120 min (Figure 6E). Images of plated samples show the final result (Figure 6F).

**Figure 7:**
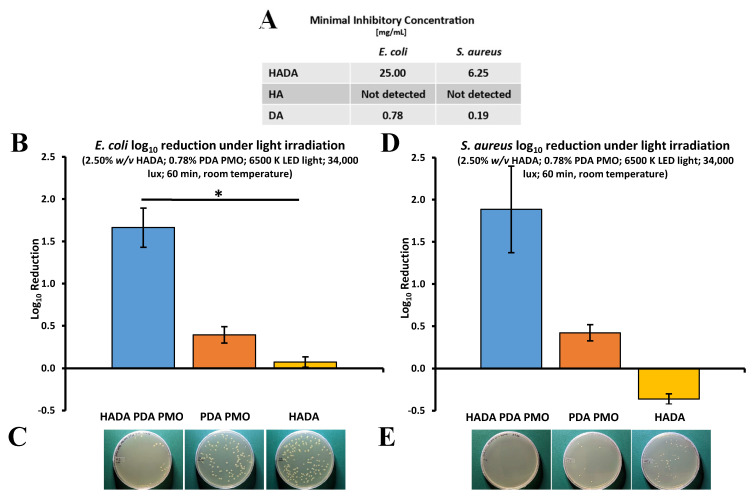
Photocatalytic antibacterial properties of HADA PDA PMO and its components against *E. coli* or *S. aureus* under visible light irradiation. (**A**) The minimal inhibitory concentration of dilutions of HADA and its components. (**B**) Nanocomposite, nanoparticles, and hydrogel effect on *E. coli* as log_10_ reduction after 60 min of visible light irradiation from LED lamp. The asterisk (*) denotes a significant difference (*p* < 0.05). (**C**) Images of *E. coli* plated samples after treatment. (**D**) *S. aureus* log_10_ reduction after similar treatment. (**E**) Images of *S. aureus* plated after treatment.

The minimal inhibitory concentration of HADA hydrogel and its components HA and DA was determined by a microdilution method using planktonic suspensions of the bacterial models. The results demonstrated that Luria–Bertani broth (LBB) solutions containing 25 mg/mL of HADA or 0.78 mg/mL of DA were effective against *E. coli*, whereas *S. aureus* was inhibited by HADA at 6.25 mg/mL and DA at 0.19 mg/mL. HA demonstrated no antibacterial effect against any of the bacterial models assessed (Figure 7A). The photocatalytic antimicrobial activity of HADA hydrogels, PDA PMO nanoparticles, and HADA PDA PMO nanocomposites under visible light irradiation produced by a commercial LED lamp was also evaluated against both bacterial strains. Results demonstrated that HADA PDA PMO nanocomposites effectively reduced the *E. coli* population by 1.66 ± 0.40 logs (94.6 ± 7.9 PK), while PDA PMO nanoparticles reached 0.39 ± 0.17 logs, and HADA hydrogels attained a nominal 0.07 ± 0.10 log_10_ reduction (Figure 7B). Images of plated samples show a visual depiction of the results (Figure 7C). In a similar fashion, a log_10_ reduction in the *S. aureus* population of 1.89 ± 0.89 (92.3 ± 7.7 PK) was obtained by HADA PDA PMO nanocomposites, with PDA PMO nanoparticles diminishing the bacterial load by 0.42 ± 0.17 logs. HADA hydrogels demonstrated no photocatalytic antimicrobial effect, with a log_10_ increase of 0.36 ± 0.11 (Figure 7D). Images of plated samples demonstrate these results (Figure 7E).

**Figure 8:**
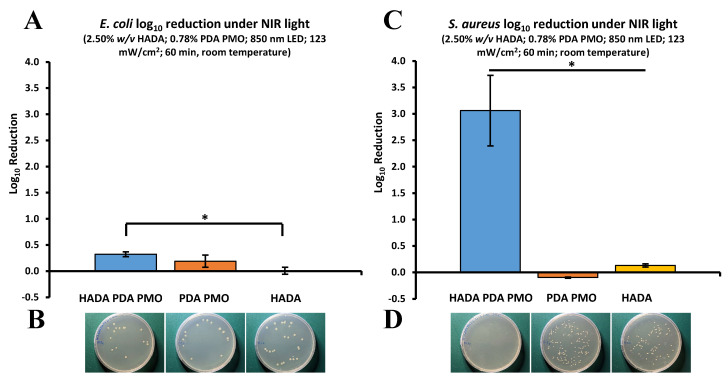
Photothermal antibacterial properties of HADA PDA PMO and its components against *E. coli* or *S. aureus* under near-infrared light irradiation. (**A**) Nanocomposite, nanoparticles, and hydrogel effect on *E. coli* as log_10_ reduction after 60 min of NIR light irradiation from an LED lamp. (**B**) Images of *E. coli* plated samples after treatment. (**C**) *S. aureus* log_10_ reduction after similar treatment. The asterisk (*) denotes a significant difference (*p* < 0.05). (**D**) Images of *S. aureus* plated after treatment.

The photothermal antimicrobial activity of HADA hydrogels, PDA PMO nanoparticles, and HADA PDA PMO nanocomposites against *E. coli* and *S. aureus* under irradiation from a commercial NIR light LED lamp was also investigated. Results for *E. coli* showed that HADA PDA PMO nanocomposites were able to reduce bacteria by 0.33 ± 0.05 logs after 60 min of exposure, while PDA PMO nanoparticles reached 0.19 ± 0.12 logs, and HADA hydrogel was ineffective at the experimental conditions (Figure 8A). Images of plated samples show the results (Figure 8B). In a similar fashion, a log_10_ reduction in the *S. aureus* population of 3.06 ± 1.15 was obtained via HADA PDA PMO nanocomposites, with PDA PMO nanoparticles showing ineffective results and HADA hydrogel exhibiting a nominal reduction of 0.13 ± 0.05 logs (Figure 8C). Images of plated samples demonstrate the final results (Figure 8D).

## 3. Discussion

In this work, we synthesized nanoparticles/nanocomposites with photocatalytic and photothermal characteristics adapting a dopamine-based in situ oxidation method [52,54] to obtain a photocatalytic metal oxide (TiO_2_) nanoparticle enveloped by a PDA coating (PDA PMO). We used titanium (IV) oxide containing two different crystalline phases of TiO_2_, anatase (80%) and rutile (20%), according to the manufacturer’s information. This mixed composition has been reported as capable to enhance the photocatalytic activity of the material [77]. Moreover, research suggests rutile also helps extend the photoactivity of anatase-rutile TiO_2_ into visible light wavelengths [78]. However, since this effect may be limited, some research groups have also used the core–shell approach to fabricate PDA coated rutile TiO_2_ nanoparticles for pollutant degradation purposes under visible light [79]. PDA by itself does not possess photocatalytic properties but can exhibit a synergistic effect with TiO_2_ and other semiconductor photocatalysts to increase the amount of π-π* electron transfer channels for an enhanced performance [27]. Furthermore, besides promoting a photocatalytic effect under visible light [54], the PDA shell also increases biocompatibility and may provide multiple chemical anchors for future nano-construction [27]. Interestingly, during PDA PMO nanoparticle synthesis, we observed a gradual change in material coloration from bright white to light brown, evidencing the formation of a PDA layer. This change in color may be due to the chemical structure or the supramolecular arrangements of PDA, as proposed by some researchers [27,80]. After this initial evidence, FTIR results confirmed the presence of peaks characteristic of the PDA coating located at around 3400 cm^−1^ (O−H in the catechol group), 1491 cm^−1^ (C=C), 1314 cm^−1^ (C−N), 1512 cm^−1^ (C=N), and 1603 cm^−1^ (C=C). The hydrodynamic size of PDA PMO nanoparticles was 262 ± 138 nm, with a polydispersity index of 0.196. Similar core–shell Ag-PDA nanoparticles have been fabricated with analogous methods, obtaining diameters around 238 nm [24].

The synthesis of mussel-inspired hyaluronic acid hydrogel (HADA) in aqueous solution resulted in 13.3 ± 1.4% dopamine content determined by UV-Vis at 280 nm, a wavelength where the absorption spectra is attributed to the phenolic groups of dopamine [27,81]. This result agrees with the 5 to 22% range reported by similar studies using EDC/NHS chemistry or any of its variants [82,83,84,85,86,87,88,89,90,91,92] and was confirmed by FTIR data showing the characteristic peaks of dopamine incorporated into the HADA spectra.

Another important effect of the PDA shell is the increase in biocompatibility it confers to TiO_2_ nanoparticles [27]. Such compatibility was demonstrated on adult human dermal fibroblasts. The compatibility study of PDA PMO suspensions at a range of 50 to 1000 µg/mL in complete media showed no adverse effect on cells after 72 h of exposure in our studies. However, since these results show elevated cell viability, they should be interpreted in the context of the tetrazolium-based MTS assay that we used. A research group recently reported [93] that DA and PDA spontaneously reduce tetrazolium in tetrazolium-based cell viability assays. They concluded that these assays may be unreliable for these specific applications and recommended the alternative use of trypan blue exclusion (TBE) to assess the cytocompatibility of dopamine-based nanomaterials, since TBE is non-dependent on redox reactions. The use of TBE to evaluate cell viability of a DA-based hydrogel has already been reported. Researchers used TBE to determine the cytocompatibility of DA-modified HA on pre-osteoblast cells dyed with Eosin Y, since the strong red fluorescence interfered with the live/dead assay originally intended [92]. Nevertheless, the TBE method also presents some issues that may affect its reliability. Besides its limited timeframe to read results due to its cytotoxic effects after five minutes of exposure [94,95,96], trypan blue also may lead to inaccuracies in cell counting, which in turn may result in cytocompatibility overestimation [97,98]. To shed some light on this conundrum, we ran a preliminary test using a TBE protocol previously published [99] to confirm the 72 h cytocompatibility of PDA PMO nanoparticles (500 and 1000 µg/mL). We reasoned that these elevated concentrations may be more likely to induce cytocompatibility issues than the lower concentrations. Results show 94.8 ± 42.4% and 75.4 ± 36.7% of cell viability, respectively. Even though these results suggest the MTS assay may overestimate the cytocompatibility by about 30%, their large standard deviation implies a great amount of variation in the groups studied and less precise data. Future work will be focused on optimizing and validating the TBE protocol to reduce variability in order to obtain reliable results.

Additionally, we studied the cell compatibility of leachates from PDA PMO nanoparticles, HADA hydrogel, and HADA PDA PMO nanocomposites prepared in deionized water (DIW). The leachates from PDA PMO nanoparticles also exhibited cytocompatibility with HDFa cells after a 72 h incubation period. Moreover, the leachates of HADA (20% *w*/*v* in DIW) and HADA PDA PMO (20% *w*/*v* HADA, 6.25% *w*/*v* PDA PMO in DIW) demonstrated cell compatibility above 80% for all dilutions. Both hydrogels and nanocomposites were formulated based on data from our previously published work on mussel-inspired nanocomposites [66,100]. The formulation was later adjusted to reflect the MIC of HADA for *S. aureus* (2x MIC, 25 mg/mL or 2.5% *w*/*v*). Thus, a nanocomposite with a reduced HADA (2.5% *w*/*v* HADA, 0.78% *w*/*v* PDA PMO) content was prepared for antimicrobial studies. Since the cytocompatibility studies were performed at higher concentrations of HADA and PDA PMO nanoparticles (8:1 ratio) than the ones used for antimicrobial studies, it is reasonable to assume that positive results from the cell compatibility studies could be extrapolated to the reduced formulation. Conversely, if no cytocompatibility is demonstrated for the higher concentration composition, then additional studies would be warranted for the reduced formulation. Considering the positive cytocompatibility results for this higher 8:1 concentration of nanocomposite, we concluded that no additional studies were needed to demonstrate the cell compatibility of the reduced formulation used for antimicrobial studies. Our biocompatibility results for hydrogels, nanoparticles, and nanocomposites concur with similar studies on PDA coated photocatalytic nanoparticles. As an example, core–shell silver polydopamine (Ag-PDA) nanoparticles showed above 95% cytocompatibility with HDF and human embryonic kidney cells at a concentration of 250 µg/mL [24]. Moreover, owning to its capacity to increase cell compatibility, PDA has been recently employed along with TiO_2_ to fabricate PDA-TiO_2_ micropatterns for selective adhesion of endothelial cells and platelets for future applications in blood-contacting devices [101]. This positive outcome also agrees with a previous report on the influence of biological coatings on the bioactivity of TiO_2_ nanoparticles [102].

Hydrogels and nanocomposites were crosslinked by a basic pH shift using sodium hydroxide (NaOH) equimolar to the DA content of the hydrogel, following methods recently published [91]. This pH-induced crosslinking of HADA hydrogels is intended to avoid the use of strong oxidizers, such as sodium periodate, that can reduce cytocompatibility [92]. This crosslinking method reportedly shows no cytocompatibility issues with NaOH-induced crosslinking of HADA hydrogels with a dopamine content of 5% and 9% [91]. In our case, the dopamine content of HADA (20% *w*/*v* in DIW) is 2.2%, and 0.3% for HADA (2.5% *w*/*v* in DIW). However, they also suggested that extensive washing steps could help to reduce the toxicity of HADA hydrogels with elevated dopamine contents, in their case, 21%. Furthermore, another research group reported the crosslinking of HADA hydrogels without the use of chemical crosslinkers [92] by using visible light (515 nm) in combination with a photosensitizer. Hence, future work will explore crosslinking strategies more apt to reduce cytocompatibility issues while maximizing compliance between the biomaterial and the targeted tissues.

The photocatalytic ability of PDA PMO nanoparticles/nanocomposites was studied by observing the degradation profile of an aqueous solution of RhB exposed to visible light. RhB is an organic dye whose photocatalytic degradation has been the subject of multiple studies using different photocatalysts. For instance, in a recent study using tungsten trioxide nanoparticles, researchers were able to use visible light to degrade up to 96.1% from a solution initially containing 5 ppm of RhB [103]. In another case, using molybdenum disulfide nanoparticles deposited onto calcium titanate, researchers attained 97% reduction from a 1 ppm RhB solution under exposure to visible light from an LED lamp (15 W, 6500 K) [104]. However, the case for TiO_2_–driven RhB degradation was limited to UV irradiation due to its lack of spectral responses under visible light. To overcome this issue, Mao et al. (2016) [54] developed an approach based on core–shell TiO_2_-PDA nanoparticles, which demonstrated total RhB degradation from an initial 10 ppm solution under visible light irradiation from a 50 W Xe lamp. A similar experiment from another group resulted in a nearly 100% RhB degradation of a 10-ppm solution under a 300 W Xe lamp, with additional tests performed on methyl orange and methyl violet solutions presenting similar results [51]. Our experimental work resulted in a 96% degradation of a 1 ppm RhB solution under light exposure from two LED lamps (10 W, 5000 K), thus confirming the potential of our PDA PMO to function as antimicrobial agents under visible light irradiation.

The photothermal antimicrobial capacity of PDA PMO is attributed to the inherent ability of PDA to work as a transducer by absorbing NIR light and transforming it into heat, with the subsequent damage to bacterial membranes [35]. In this regard, PDA has been explored as a minimally invasive platform for photothermal therapy to ablate cancer cells without damaging healthy tissues [36,105,106,107,108]. It also has been investigated for wound healing [40] and anti-bacterial photothermal therapies [35]. As reported by some researchers, experimental work has been conducted at controlled photo-induced temperatures (<45 °C) with the expectation of high lethality against bacterial species and minimal damage to surrounding tissue. However, due to the potentially uneven local heat distribution resulting from the PDA coating, the antibacterial efficacy might be reduced and the side effects on normal cells could be increased. Hence, this strategy requires extended research to determine its clinical feasibility.

On the other hand, the photocatalytic antibacterial properties of PDA PMO nanoparticles/nanocomposites result from ROS formation due to the absorption of photons and the subsequent creation of excited-state electron-hole pairs [46,109]. Due to its semiconductive properties, titanium dioxide is able to continuously adsorb photons from ultraviolet (UV) light, resulting in the promotion of electrons from the valence to the conduction band. This in turn creates positive charged free spaces called valence band holes. Since holes and electrons can migrate, they can recombine unproductively, or they can journey to the surface for further reactions. This effect can be extended into visible light by the action of a PDA coating [54], which modifies the surface of TiO_2_ to improve its photocatalytic capacity by increasing visible light absorption and transfer of photogenerated electrons while decreasing the recombination rate [27,110]. After reaching the surface, electrons and holes react with environmental moisture or oxygen to produce hydroxyl radicals (⋅OH), superoxide ions (O_2_^−^), or hydrogen peroxide (H_2_O_2_), among other ROS [47,49,111,112]. Previously published literature [45] suggests that superoxide (O_2_^−^) is the main ROS generated by PDA-coated TiO_2_ under light irradiation and is formed by the capture of an electron in a π*2*p* orbital of oxygen [47]. However, these long-lasting superoxide ions are not very toxic to microorganisms since they cannot penetrate the bacterial membrane due to their negative charge, but they can present additional reactions to form hydrogen peroxide (H_2_O_2_) and hydroxyl radicals (⋅OH) [47,49]. Studies on the antimicrobial effect of TiO_2_ have shown that free and surface bound ⋅OH radicals are the major contributors to its antimicrobial effect, with O_2_^−^ and H_2_O_2_ as minor players [113].

Hydroxyl radicals reportedly have a very short life-span (10 × 10^−9^ s) [114], which limits their ability to diffuse far from the surface. Hence, their ability to kill bacteria can only be exerted at or in the vicinity of the nanoparticle surface. Typically, microorganisms can deal with less reactive superoxide and hydrogen peroxide using endogenous antioxidants, such as superoxide dismutase and catalase [115,116], but hydroxyl radicals may be lethal [47,48,111] since they are known to be highly reactive towards any organic compound inside the microbe [115]. Moreover, ⋅OH radicals cause the loss of microbial membrane integrity, as demonstrated by Gogniat et al. (2006) [114]. In turn, the secondary contributors of H_2_O_2_ and O_2_^−^ ions live longer and may present antimicrobial effects at a longer distance from the nanoparticles/nanocomposites. For instance, hydrogen peroxide can pass through the bacterial wall [117] to react with ferrous ions resulting in the production of detrimental ⋅OH radicals. Furthermore, extracellular H_2_O_2_ and O_2_^−^ are highly reactive to biomolecules and also are precursors for additional ⋅OH radicals [117]. Additionally, an indirect antibacterial mechanism may be due to the self-propagating lipid peroxidation after ROS reaction with fatty acids in the bacterial membrane [47,118]. Moreover, the presence of hydrogen peroxide may also induce a bacteriostatic effect, as previously demonstrated by a research group who found PDA was able to inhibit the growth of *E. coli* on cetacean skin due to the release of micromolar amounts of H_2_O_2_ [119].

In addition to the photocatalytic and photothermal capacities of PDA PMO, HADA exhibits some antimicrobial effect as shown by minimal inhibitory concentration studies. It was found that HADA and DA solutions were able to kill both bacterial models, but hyaluronic acid demonstrated no antibacterial effect at all. Furthermore, cultures of samples on or above MIC values presented no bacterial growth, confirming the bactericidal effects of HADA and DA. These results may be attributed to dopamine, which has been the subject of previous experimental work that demonstrates its antimicrobial effect against *E. coli* [120] and *S. aureus* [121]. When exposed to visible light under similar experimental conditions, PDA PMO and HADA PDA PMO presented killing efficacies within the same range value, confirming PDA PMO as the material responsible for their antimicrobial effect. However, the main mechanism or combination of mechanisms to which this effect can be attributed is still to be elucidated. Furthermore, under NIR light HADA PDA PMO presented an adequate killing efficacy for *S. aureus* but underperformed on *E. coli*.

According to the information we have found, HADA PDA PMO is a novel nanocomposite reported here for the first time. In a similar manner, nanocomposites with photo-responsive properties for antimicrobial purposes have been developed based on hyaluronic acid and silver nanoparticles for wound healing purposes. In one case, researchers developed an antimicrobial/antioxidant nanocomposite to reduce bacterial colonization based in hyaluronic acid-tyramine hydrogel combined with silver nanoparticles capped with tannic acid. The material was assessed on in vivo models of skin infection irradiated with NIR light (808 nm, 920 mW/cm^2^) with results showing 100 PK after 10 min of irradiation [122]. Another study using a chitosan-based composite film loaded with silver nanoparticles conjugated with carbon nitride-polydopamine was tested in vitro against *S. aureus* and *P. aeruginosa* under visible light irradiation for 10 min, with results showing bacterial killing ratios of 100% [41]. Moreover, other metals have also been used to fabricate photocatalytic antimicrobials. Such is the case of copper-added organic molecules, which under the irradiation of visible light, demonstrated 99.7% efficacy to kill *S. aureus* due to ROS and heat generation [123]. The publications from these researchers are an example of the importance of alternative approaches for infection treatment, in order to prevent antibiotic-resistance development. Fields such as wound healing, infection prevention, and orthopedics can benefit from the development of multiple photocatalytic or photothermal materials [124].

Among the limitations of our current work, the synthetic route to prepare HADA presents some constraints that impede the conjugation efficiency of catechol groups, probably resulting from the oxidative polymerization of dopamine as a result of oxygen dissolved in the aqueous media. One way to overcome this issue is the use of an alternative synthetic route based on the preliminary oxidation of HA to form dialdehyde groups followed by Schiff base reaction with dopamine, as demonstrated by Zhou et al. (2020) [125], who reported degrees of substitution as high as 45%. This greater grafting efficiency may be the consequence of a higher reactivity of dialdehyde groups as compared to carboxylic acid groups in EDC/NHS coupling. Hence, the use of improved HADA synthesized via Schiff base reaction may be beneficial since a higher dopamine content might enhance the antimicrobial properties of the hydrogel. Similarly, another area of improvement is the optimization of HADA PDA PMO formulations to increase their killing efficacy up to the levels reported by other approaches [41,122]. Moreover, current PDA PMO nanoparticle synthesis presents ample room for improvement to increase their visible light adsorption and electron/hole separation capacity, which are a function of PDA coating thickness, as suggested in the literature [54]. Additionally, PDA PMO might be self-limiting its antimicrobial effect since PDA is also known as a ROS scavenger [126,127]. Hence, it is reasonable to expect some ROS self-scavenging activity from the PDA coating, but measuring any detrimental effect on the overall performance of PDA PMO is beyond the scope of this work. However, the experimental evidence suggests that even if this activity is happening, its effect is not hindering the antimicrobial activity of PDA PMO. Furthermore, to the best of our knowledge, the mechanism and kinetics of any potential ROS self-scavenging activity by PDA are still not well understood or reported. Moreover, in future work, this self-scavenging interaction might be mitigated by the inclusion of TEMPO (2,2,6,6-tetramethylpiperidine 1-oxyl radical), which possesses a strong synergy with PDA [27,128], has the ability to increase the photocatalytic efficiency and antimicrobial effect, and has been used to prepare biodegradable nanocomposites based in sodium alginate [129]. All experiments in this work were designed to evaluate antimicrobial performance under light exposure, but because TiO_2_ nanoparticles reportedly present inherent antibacterial activity [111] and can limit bacterial growth in the dark [130], additional work is warranted to evaluate the antimicrobial capacity in the absence of light.

## 4. Materials and Methods

### 4.1. Materials

Sodium hyaluronate (MW 150,000–300,000 Da) was supplied by Lifecore Biomedical. Additionally, 1-ethyl-3-(3-dimethylaminopropyl) carbodiimide (EDC), Titanium (IV) oxide (TiO_2,_ Cat. Nr. 718467), N-hydroxy succinimide (NHS), and Dopamine hydrochloride (DA) were obtained from Sigma, whereas Alfa Aesar supplied hexamethylenetetramine (HMTA) and Acros Organics 4-Morpholineethanesulfonic acid (MES). All materials were used as received.

### 4.2. Elaboration of Photo Responsive Nanoparticles

Photocatalytic nanoparticles (PDA PMO) were fabricated by coating TiO_2_ nanoparticles with a thin film of polydopamine, adapting methods previously published in the literature [52,54]. In brief, TiO_2_ was resuspended in 0.22 µm filtered DIW (450 mL, 2.66 mg/mL), vortexed, and sonicated (Q700, QSonica LLC) with a 0.5 in probe (20 min active time, 60 s on/30 s off, 40% amplitude) until the particle size assessed by dynamic light scattering (DLS) (NanoBrook 90Plus PALS, Brookhaven Instruments Co., Holtsville, NY, USA) was around 200 nm. Then, the suspension was transferred to a 1000 mL flask, placed into an oil bath, and mixed at 700 rpm. After the temperature reached 90 °C, 150 mL of DA solution (0.948 mg/mL in filtered DIW) and 150 mL of HMTA solution (9.95 mg/mL in filtered DIW) were added. The reaction proceeded in the dark for 3 h. Then, PDA PMO was recovered by centrifugation (5430 R, Eppendorf, 7000 rpm, 5 min, 20 °C), washed two times with DIW, one time with ethanol 70%, and lyophilized.

### 4.3. Characterization of Photo Responsive Nanoparticles

The PALS technique (NanoBrook 90Plus PALS, Brookhaven Instruments Co.) was used to measure the zeta potential while hydrodynamic size was determined by DLS. Nanoparticles (0.01 % *w*/*v*) were resuspended in phosphate-buffered saline (PBS, pH 7.2) to perform PALS and in DIW for DLS. To confirm the presence of a PDA coating film on TiO_2_ nanoparticles, FTIR spectroscopy (FTIR, Nicolet 6700, Thermo Fisher Scientific Inc., Waltham, MA, USA) analysis of freeze-dried nanoparticles was performed. For imaging and composition confirmation, simultaneous EDS and SEM were performed (Hitachi S-3000 N). EDS/SEM samples were prepared by adding 0.5 mg of freeze-dried PDA PMO onto carbon tape and observed under SEM at a 25 kV acceleration setting and a magnification of 18K. No sputter coating with conductive material was required for SEM imaging/EDS analysis because of the metallic nature of these nanoparticles. Morphological images of PDA PMO were also acquired via HR-TEM (Hitachi H-9500 FE-HRTEM). A suspension of PDA PMO in DIW (0.05% *w*/*v*, 10 µL) was cast onto a support grid (FF200-Cu, Electron Microscopy Sciences) and then visualized at a 40 kV acceleration setting. The photocatalytic activity of PDA PMO was evaluated as a function of RhB degradation under visible light at room temperature (RT), adapting methods previously published [52,54,131,132,133,134]. In brief, 20 mL of a suspension containing RhB (1 ppm) and PDA PMO (2 mg/mL in filtered DIW) was placed in a glass scintillation flask and stirred at 300 rpm. Then, the flask was exposed to illumination from two 10 W LED lamps (5000 K, 34,000 lux). The luminous flux per unit area (lux) was measured using a digital light meter (LX 1330B, Dr. Meter). A total of 500 µL samples were taken at predetermined timepoints and centrifuged (13,000 rpm, 20 min) to sediment the nanoparticles. Thereafter, 150 µL (*n* = 3) of supernatant was placed into a 96-well plate to run an absorbance scan. Bleach 10% (200 µL) was a positive control. RhB degradation was calculated from time-point absorbance values at 555 nm (*At*) compared with the initial absorbance (*Ao*) to calculate % RhB degraded as [(*Ao* − *At*)/*Ao*] × 100.

### 4.4. HADA Synthesis

Hyaluronic acid–dopamine was synthesized via EDC-NHS chemistry in an aqueous solution at RT and a dark setting according to the literature [66,86]. Briefly, hyaluronic acid (HA) was dissolved in MES buffer (30 mL, 10 mg/mL, pH 6.2, 300 rpm). Then, nitrogen (5 psig, 30 min) was injected and bubbled under the same mixing conditions to deoxygenate the solution. Next, after the addition of EDC (493 mg) and NHS (291 mg), the reaction under a nitrogen blanket continued for 30 min followed by the addition of a solution of dopamine hydrochloride (DA, 2 mL, 250 mg/mL) in MES buffer (pH 6.2). After 12–16 h, the reaction was stopped and HADA was purified by dialysis (3500 Da MWCO or membrane molecular weight cut-off, 24 h) in cold DIW (4 °C). Afterwards, the material was dried by lyophilization. To confirm the conjugation of DA on the backbone of HA, the chemical composition was analyzed using FTIR(Nicolet iS50, Thermo Fisher Scientific Inc.). The DA content was calculated via UV-Vis (Infinite M200, Tecan, Männedorf, Switzerland) measuring the 280 nm absorbance values in the linear range of a HADA sample (1 mg/mL, *n* = 3) compared to a DA standard curve (0–1 mg/mL range, *n* = 3).

### 4.5. Formulation and Characterization of Hydrogel and Nanocomposites

A composition of 20% *w*/*v* HADA and 6.25% *w*/*v* PDA PMO was used for the degradation and cytocompatibility studies. HADA hydrogel (20% *w*/*v* in DIW) and HADA PDA PMO nanocomposite (20% *w*/*v* HADA, 6.25% *w*/*v* PDA PMO in DIW) were formulated and crosslinked with NaOH 1N equimolar to the DA content of HADA. In brief, the desired amount of PDA PMO was resuspended in the aqueous phase and sonicated to break down any agglomerates. Then, HADA was added in small portions, and the mixture was vortexed until total dissolution of HADA, followed by the addition of sodium hydroxide 1 N. After vortexing, the materials were allowed to crosslink for 60 min. To prepare samples for degradation and cytocompatibility studies, the materials were used in bulk, with no shaping nor additional purification processes. To observe the degradation profile of HADA and HADA PDA PMO, samples were prepared as described above. Then, 50 mg of crosslinked materials were submerged (*n* = 3) in 0.5 mL of bovine hyaluronidase solution (100 units/mL in PBS). At time-points 6, 24, 48, and 72 h, the samples were centrifuged (5430 R, Eppendorf, 17,000 rpm, 10 min, 20 °C) to collect any undegraded material, which then was rinsed with pure ethanol and air dried to measure its dry weight (W_t_). The remaining weight was estimated as % W*_r_* = W*_t_*/W*_o_* × 100%, where W*_r_* is the remaining weight, W*_t_* the weight at timepoint *t*, and W*_o_* is the initial weight. To study the cytocompatibility of HADA and HADA PDA PMO leachates, 100 mg of crosslinked HADA (20% *w/v* in DIW) or HADA PDA PMO (20% *w*/*v* HADA, 6.25% PDA PMO in DIW) were incubated with 1 mL of complete media for 72 h, as described in Section 4.6.

After performing MIC studies, the formulation was adjusted to reflect the MIC of HADA for *S. aureus* (2× MIC, 25 mg/mL or 2.5% *w*/*v*). Thus, a nanocomposite with a reduced content of HADA (2.5% *w*/*v* HADA, 0.78% *w*/*v* PDA PMO) was prepared for antimicrobial studies. Since the cytocompatibility studies were performed at higher concentrations of HADA and PDA PMO nanoparticles (8:1 ratio) than the ones used for antimicrobial studies, it is reasonable to assume that positive results from the cell compatibility studies could be extrapolated to the reduced formulation. Conversely, if no cytocompatibility is demonstrated for the higher concentration composition, then additional studies would be warranted for the reduced formulation.

The morphology of hydrogel and nanocomposites was examined using SEM. In brief, HADA hydrogel (2.5% *w*/*v* in DIW) and HADA PDA PMO nanocomposite (2.5% *w*/*v* hydrogel, 0.78% *w*/*v* nanoparticles in DIW) were formulated as previously described and left for 60 min to swell. Then, 10 µL samples were pipetted onto glass slides, inserted in 50 mL tubes for protection, and placed in a −80 °C freezer overnight. The frozen samples were then lyophilized and prepared for SEM imaging using a Hummer VI sputtering system. The samples were coated in Gold (Au)/Palatium (Pl) deposition for 1.5 min. After coating, samples were detached from the glass slides and placed on a metal SEM sample holder using double sided carbon tape. Then, images of the samples were taken with a Hitachi S-4800 II FE-SEM. The conjugation of carboxyl groups of hyaluronic acid with amine groups of dopamine was assessed by examining dry samples of HADA using FTIR (Nicolet 6700, Thermo Fisher Scientific Inc.).

### 4.6. Cytocompatibility Studies

The cytocompatibility of PDA PMO suspensions was evaluated on adult human dermal fibroblasts (HDFa, ATCC) following methods previously published [66,100]. In brief, sterile nanoparticle suspensions (50, 100, 500, and 1000 µg/mL) were prepared in complete media formed using Dulbecco’s Modified Eagle Medium (DMEM, Sigma Aldrich, St. Louis, MO, USA), with added FBS (fetal bovine serum, 10% *v*/*v*, Corning Inc., Corning, NY, USA) and Pen-Strep (penicillin-streptomycin, 1% *v*/*v*, Life Technologies Corporation, Carlsbad, CA, USA). Concurrently, 96-well plates were used to seed HDFa (*n* = 6) at 10,000 cells/well followed by incubation (24 h, 37 °C, 5% CO_2_) in complete media. Then, media was replaced with 150 µL/well of nanoparticle suspensions, followed by incubation for additional 72 h. Thereafter, the cell viability was assessed with an MTS assay (CellTiter 96, Promega Co., Madison, WI, USA) following the company’s instructions. Triton X-100 (1% *v*/*v*) was used as a negative control, whereas cells treated with complete media served as positive control. Results were also confirmed by determining the cell compatibility of PDA PMO leachates using a method adapted from the literature [135]. In short, 100 mg of PDA PMO were incubated with 1 mL of complete media during 72 h at 37 °C, followed by sterilization by filtration with 0.2-micron filter and subsequent preparation of 1×, 10×, and 100× leachate dilutions in complete media. Simultaneously, HDFa were seeded in 96-well plates (*n* = 6), as described above, and incubated for 24 h in 200 µL of complete media. Thereafter, 20 µL of PDA PMO leachate dilutions were added to their respective wells and incubation continued for 72 h. Afterwards, the cell viability was assessed as previously described. Similarly, HADA hydrogel and HADA PDA PMO nanocomposite leachates were evaluated. Briefly, 100 mg of crosslinked HADA (20% *w*/*v* in DIW) or HADA PDA PMO (20% *w*/*v* HADA, 6.25% PDA PMO in DIW) were incubated with 1 mL of complete media for 72 h. Thereafter the leachates were collected and sterilized by filtration with a 0.2-micron filter followed by preparation of 1×, 10×, and 100× leachate dilutions in complete media. HDFa were seeded and incubated in 96-well plates (*n* = 6), as described previously, with the subsequent addition of 20 µL of leachate dilutions and posterior incubation for 72 h. Then, the cell viability was assessed by MTS assays. Additionally, cells were imaged in the contrast phase (ECHO Revolve, Discover Echo Inc., San Diego, CA, USA) at the end time points.

### 4.7. Antimicrobial Studies

The photocatalytic antimicrobial performance of PDA PMO and TiO_2_ was compared by challenging samples of both materials with strains of *S. aureus* (Wichita strain, ATCC Catalog No. 29213) or *E. coli* (K12 strain, Carolina Biosciences Catalog No. 155065). The test was conducted under visible light irradiation (6500 K LED light; 34,000 lux) for 120 min at room temperature (RT) using a method adapted from the literature [136]. In short, plastic Petri dishes (35 mm diameter) were coated with sterile PDA PMO or TiO_2_ suspensions at a density of 0.2 mg/cm^2^ and left to dry overnight. Untreated Petri dishes were used as controls. Next, a bacterial suspension in LBB was prepared using the direct colony suspension method, in which three to five colonies are picked up from a plate previously streaked with frozen bacteria culture to separate individual colonies. The collected colonies are then resuspended via vortexing, and the optical density 600 (OD_600_) of the bacterial suspension is set to a value between 0.08 and 0.13. Once OD_600_ was adjusted to the desired level, the bacterial suspension was further diluted 1:10 in LBB to prepare diluted culture. Then, a test suspension was made by mixing 340 µL of diluted culture with 660 µL of LBB. Subsequently, 40 µL of test suspension were dropped into coated and uncoated Petri dishes and distributed uniformly using sterile plastic loops (2 µL). The dishes were sealed, placed under illumination, and kept at RT via direct air circulation. After 2 h, the samples and controls were removed from under the LED lamp and rinsed two times with 1 mL each of phosphate-buffered saline (PBS). Rinsing fluid was collected and further diluted 1:10 in PBS. Then, 100 µL (*n* = 3) was plated in LBA and incubated for 18–24 h at 37 °C. Thereafter, the number of colony-forming units (CFU) present in each plate was counted manually or using Image J 1.53 (U.S. National Institutes of Health) if they were TNTC (too numerous to count, >200 CFU). The log_10_ reduction (LR) of bacterial populations was estimated following methods described in the literature [137]. The kinetics of the photocatalytic activity of PDA PMO against *S. aureus* or *E. coli* was tested under similar conditions and following the method described above.

A qualitative determination of MIC, the minimal concentration of HADA required to inhibit bacterial growth, was performed using a microdilution method adapted from the literature [138,139,140,141]. In brief, serial dilutions (0.02–25 mg/mL) of HADA, HA, and DA in LBB were arranged in a 96-well plate (*n* = 4). Then, using the procedure described above, a bacterial suspension with an OD_600_ between 0.08 and 0.13 was prepared and used to formulate a standardized inoculum containing about 5 × 10^5^ CFU/mL. Next, 50 µL of inoculum/well were added, and the plate was incubated (16 h, 37 °C). Thereafter, 30 µL of resazurin (0.015% in DIW) were added to each well and incubation continued for 2 more hours. At the conclusion of the experiment, wells that remained blue were scored as above the MIC value, and their content was plated in Luria–Bertani agar (LBA) plates to determine the Minimum Biocidal Concentration (MBC). Growth control and sterility control wells were included in the experiment.

The antimicrobial activity of HADA PDA PMO nanocomposite and its components against *S. aureus* or *E. coli* was determined under light exposure (6500 K LED light; 34,000 lux) for 60 min by further adapting the method mentioned above. Briefly, 12-well plates were coated (*n* = 3) with 50 µL/well of HADA PDA PMO (2.5% *w*/*v* HADA, 0.78% *w*/*v* PDA PMO), HADA (2.5% *w*/*v*), or PDA PMO (0.78% *w*/*v*) prepared as previously described. Then, a bacterial suspension of OD_600_ between 0.06 and 0.09 was prepared and diluted to make a test suspension calibrated to reach a survival bacterial count between 4 to 5 logs in the untreated (negative) control [136], as described above. Thereafter, each well was inoculated with 40 µL of test suspension, and the inoculum was distributed uniformly on the well surface. The test plate was placed under light irradiation for 60 min followed by vigorously washing each well two times with 1 mL PBS each. The wash was collected and diluted 1:10 in PBS. Thereafter, 100 µL (*n* = 3) was plated in LBA. Plates were incubated for 18–24 h to count CFUs and calculate LR as described above. Negative control (no treatment) was LBB.

To further explore the antimicrobial properties of HADA PDA PMO nanocomposite and its components, the method was adapted for near-infrared (NIR) light irradiation (850 nm, 123 mW/cm^2^). In brief, 12-well plates were coated (*n* = 3) with HADA PDA PMO, HADA, or PDA PMO and inoculated via test suspension, as described above. Then, the plate was placed under NIR light irradiation for 60 min followed by washing, plating, and incubation as mentioned above. CFU counting and LR calculations were performed as previously described. Negative control was LBB.

### 4.8. Statistical Analysis

Significant differences (*p* < 0.05) among experimental groups were assessed with one-way ANOVA. Post hoc analysis was executed by Tukey’s HSD (Honest Significant Difference) test. Data were reported as mean ± standard error of the mean.

## 5. Conclusions

In this proof-of-concept experimental work, dual photo-responsive nanoparticles were prepared using oxidative polymerization of dopamine to synthesize a polydopamine coating onto a photocatalytic metal oxide core. The resulting core–shell PDA PMO nanoparticles demonstrated their photocatalytic potential by oxidizing aqueous solutions of Rhodamine-B under visible light irradiation from commercial LED lamps. Moreover, when compared to TiO_2_ nanoparticles, PDA PMO demonstrated superior photocatalytic antimicrobial performance against planktonic suspensions of *E. coli* and *S. aureus* under visible light. After 120 min of LED light exposure, PDA PMO significantly reduced bacterial counts of both species. Furthermore, the mussel-inspired hydrogel HADA also demonstrated antimicrobial activity on its own. The synergy of PDA PMO and HADA was further explored by combining them into the mussel-inspired nanocomposite HADA PDA PMO, which exhibited important antibacterial properties triggered by visible light. Furthermore, the exposure of HADA PDA PMO to near-infrared light resulted in a significant photothermal antimicrobial effect against *S. aureus*. Hence, these results suggest the potential use of PDA PMO and HADA PDA PMO in diverse fields of bioengineering and translational medicine to meet the demands of applications as diverse as NIR-driven wound healing treatment or prevention of catheter infection using visible light.

## Data Availability

The data that support the findings of this study are available from the corresponding author upon reasonable request.
